# Impact of the COVID-19 pandemic on antibiotic treatment for respiratory tract infections in Norwegian primary care

**DOI:** 10.1080/02813432.2026.2617522

**Published:** 2026-01-19

**Authors:** Leo Larsen, Valborg Baste, Guri Rortveit, Knut Erik Emberland

**Affiliations:** a Department of Global Public Health and Primary Care, University of Bergen, Bergen, Norway; b National Centre for Emergency Primary Health Care, NORCE Norwegian Research Centre, Bergen, Norway; c Director General’s Office, Norwegian Institute of Public Health, Oslo, Norway

**Keywords:** Respiratory tract infections, antibiotics, primary health care, COVID-19, pandemic

## Abstract

**Background:**

During the COVID-19 pandemic, the use of macrolides, specifically azithromycin, for respiratory tract infections (RTIs) in primary care increased in several countries. In Norway, antibiotic treatment of COVID-19 was never recommended.

**Objectives:**

To investigate the antibiotic treatment for RTIs in Norwegian primary care, comparing pre-pandemic and pandemic periods.

**Methods:**

We defined RTI episodes and antibiotic treatment using several national registries including demographic and residency data from Statistics Norway, reimbursement claims from the Norwegian Registry for Primary Health Care, antibiotic dispensing from the Norwegian Prescription Database, and deaths from the Norwegian Cause of Death Registry, for the years 2018–2021.

**Results:**

Approximately 80% of the 4 904 376 total RTI episodes during the study period were handled exclusively in daytime general practice (DGP). Use of electronic consultations for RTI episodes increased from less than 1% to more than 50%. Throughout the study period, most RTI episodes were handled without antibiotic use. The antibiotic treatment rate for RTI episodes dropped during the pandemic, relative risk (RR) 0.52, 95% confidence interval (CI) 0.52–0.52, compared to pre-pandemic. Over half of all antibiotic treatments for RTIs were phenoxymethylpenicillin, and the distribution of antibiotic types was relatively stable during the study period, except for some temporary changes in the initial months of the pandemic. DGP handled most of the influx of RTIs during the first month of the COVID-19 pandemic in primary care, without increasing antibiotic use.

**Conclusions:**

DGP *handled* most of the influx of RTIs during the initial phase of the COVID-19 pandemic in primary care. During the pandemic antibiotic treatment for RTIs was reduced, and the distribution of antibiotic types barely changed.

## Introduction

Antimicrobial resistance is a major threat to health and health care systems globally [1]. Prescribing antibiotics in primary care is linked to increased antibiotic resistance on both population and individual level [2–4]. In Norway, 84% of antibiotics for humans are prescribed in primary care [5]. The proportion of respiratory tract infections (RTIs) with antibiotic prescriptions in primary care in Norway decreased from 37% in 2012 to 23% in 2019 [6]. Still, a 2020 study from Sweden, a comparable country, shows RTIs remain the most common reason for primary care antibiotic use [7].

Norway declared the first national intervention against the spread of SARS-CoV-2 on 12 March 2020, consisting of a major societal lockdown. On 17 March 2020, the government urged health care services to use remote video consultations to reduce the spread of COVID-19 [8]. There was also a municipal pandemic response, including establishing separate infection wards in emergency primary care services, municipal COVID-19 wards, and separate airway clinics [9].

A study of 16 European countries found a mean reduction in primary care antibiotic prescriptions for RTIs, from 31.6% before the pandemic to 17.6% during the early stages of the pandemic [10]. We are not aware of similar studies in a Norwegian population, but the sales of systemic antibiotics in Norway dropped sharply after March 2020, with the most significant reductions observed in sales of antibiotics commonly used to treat RTIs [11]. However, the study did not have access to data on whether the antibiotics were prescribed specifically for RTIs.

Early in the pandemic, there was some uncertainty regarding the treatment of COVID-19, and azithromycin was discussed as a potentially beneficial drug in this regard [11,12]. In England, azithromycin was not promoted as treatment for COVID-19. However, there was increased prescribing of azithromycin for RTIs in 2020 compared to 2019, and an increase in COVID-19 cases correlated with higher rates of azithromycin prescription [13]. In Norway, there were no guidelines promoting the use of antibiotics for COVID-19 [11].

Knowledge about the initial and sustained effect of the pandemic on health care services and antibiotic treatment is crucial in emergency preparedness planning and can help guide interventions in work to reduce the unnecessary use of antibiotics.

### Aims

The aim of this study was to investigate the impact of the COVID-19 pandemic on antibiotic treatment for RTIs during the pandemic in Norwegian primary care.

## Methods and materials

### Primary care in Norway

All residents in Norway are entitled to a general practitioner (GP), through daytime general practice (DGP) or out-of-hours (OOH) services. GPs in Norway function as the primary contact point for patients with the health care services. Secondary health care services are normally only accessible through referral from primary health care (DGP or OOH services). A prescription is needed to obtain antibiotics, and all dispensing is registered. There are national guidelines for antibiotic treatment, both for primary and secondary care [14]. The International Classification of Primary Care version 2 (ICPC-2) is the mandatory diagnostic coding system for DGP and OOH services in Norway.

### Data sources

We used national registry data from 2018 to 2021: demographic and residency data from Statistics Norway (SSB), primary care reimbursement claims from the Norwegian Registry for Primary Health Care (NRPHC), community pharmacy dispensing of antibiotics from the Norwegian Prescribed Drug Registry (NorPD), and deaths from the Norwegian Cause of Death Registry (NCDR).

### Study population

We included all registered reimbursement claims from in-person and electronic consultations in DGP or OOH services in the years 2018–2021 with an RTI diagnosis code. We did not include simple administrative contacts.

Data from all consultations for respiratory tract infections from the NRPHC were linked by the patients’ pseudonymous unique personal identifiers to demographic data from the source SSB data set and to data on antibiotic treatments from NorPD.

We used residency data from SSB and time of death from NCDR to generate monthly population counts by age group and sex. For 2021, the SSB registry data was not updated with newborn, immigrated, or emigrated persons.

### RTI consultation and RTI episode

We defined an RTI consultation as a registration in NRPHC containing at least one reimbursement code for an in-person or electronic consultation, combined with at least one ICPC-2 code indicating an RTI (Supplementary Table 2). We based the definition on the one used in Larsen et al. [15], with some modifications.

We grouped RTI consultations into RTI episodes to account for multiple consultations for the same RTI. An RTI episode was defined as an index RTI consultation with no RTI consultations in the preceding 30 days. Follow-up RTI consultations in the same episode, were defined as RTI consultations that occurred within 30 days of the preceding RTI consultation, irrespective of whether the preceding RTI consultation was an index or follow-up consultation. We chose this time limit to include re-consultations for the same infection, and any follow-up after, similar to previous works [6,16–18]. We did not set a maximum episode length or limit the number of consultations per RTI episode.

### Antibiotic treatment

We included data on standard prescription dispensing of the following types of oral antibiotics from the Anatomical Therapeutic Chemical Classification System (ATC) chapter J01: phenoxymethylpenicillin (J01CE01), other penicillins (other J01C), tetracyclines (J01A), macrolides (J01FA), and other antibiotics (other J01, vancomycin (A07AA09), and metronidazole (P01AB01)). We excluded medications where their only indication was treatment of urinary tract infections: pivmecillinam (J01CA08), mecillinam (J01CA11), trimethoprim (J01EA01), nitrofurantoin (J01XE01) and methenamine (J01XX05).

We defined an ‘RTI consultation with antibiotic treatment’ as an RTI consultation followed by the dispensing of an antibiotic within 7 days, to account for pharmacies closing on holidays and other potential delays in prescription filling, and is in line with previous studies [6,16,19]. We defined an ‘RTI episode with antibiotic treatment’ as an RTI episode containing at least one RTI consultation with antibiotic treatment. We did not set a maximum number of antibiotic treatments per RTI episode.

### Variables

We defined the outcome as antibiotic treatment for RTIs. The exposure was the COVID-19 pandemic period. We defined 12 March 2020 as the start of the pandemic period. In analyses comparing the pre-pandemic and pandemic period we used time periods of similar length for more representative comparisons (12 March 2018 to 30 November 2019 and 12 March 2020 to 30 November 2021). We excluded January 2018 and December 2021 from the analysis in Table 2, due to the temporal aspect of episode and antibiotic treatment definitions.

**Table 2.** Respiratory tract infection episodes and antibiotic treatment before and during the COVID-19 pandemic (*N* = 3869712).

	Pre-pandemic		Pandemic			
	No. of episodes	% with antibiotic treatment	No. of episodes	% with antibiotic treatment	Risk ratio	95% CI
Patient age						
0–4	310384	21.1	200348	14.9	0.71	0.70–0.72
5–14	192965	24.0	179097	9.7	0.40	0.40–0.41
15–24	357801	24.8	292241	16.1	0.65	0.64–0.65
25–34	263420	26.1	314271	11.2	0.43	0.43–0.44
35–44	226333	27.3	272171	11.4	0.42	0.41–0.42
45–54	213818	26.5	227809	11.9	0.45	0.44–0.45
55–64	189547	29.0	172006	14.8	0.51	0.50–0.52
65–74	151990	32.0	107164	18.9	0.59	0.58–0.60
75–84	84289	31.7	59055	20.0	0.63	0.62–0.64
85+	33520	32.6	21483	20.9	0.64	0.62–0.66
Total	2024067	26.1	1845645	13.5	0.52	0.52–0.52
Service type*						
DGP (1)	1418207	19.4	1102172	9.3	0.48	0.48–0.48
OOH (1)	210610	29.3	233232	14.6	0.50	0.49–0.50
DGP (2+)	287219	46.1	358922	18.4	0.40	0.40–0.40
OOH (2+)	14016	47.0	19239	28.1	0.60	0.58–0.62
Mixed (2+)	94015	56.5	132080	31.1	0.55	0.55–0.56
Consultation mode*						
In-person (1)	1619923	20.7	764720	13.3	0.64	0.63–0.64
Electronic (1)	8894	5.9	570684	6.2	1.06	0.97–1.15
In-person (2+)	382737	48.9	121389	34.5	0.71	0.70–0.71
Electronic (2+)	663	10.9	167068	8.2	0.76	0.61–0.94
Mixed (2+)	11850	39.6	221784	25.7	0.65	0.63–0.66

* Parentheses indicate number of consultations in episode.

Mantel-Haenszel test for heterogeneity, *p*-values for differences in patient age: < 0.0001; service type: < 0.0001; consultation mode: < 0.0001.

CI: Confidence interval; DGP: Daytime general practice; OOH: Out-of-hours.

### Service type and consultation mode

Service type for each RTI consultation was recorded as either ‘daytime general practice’ or ‘out-of-hours’ in the NRPHC data. We defined consultation mode as either ‘in-person’ or ‘electronic’ based on procedure codes. RTI episodes were labelled likewise, except for ‘mixed’ RTI episodes containing consultations of differing service type or consultation mode.

### Demographic variables

Demographic variables were age and sex. We grouped patient age as follows: 0–4, 5–14, 15–24, 25–34, 35–44, 45–54, 55–64, 65–74, 75–84 and ≥85 years.

### Statistical analysis

Data processing, analysis and visualisation was done using Stata/SE version 18.5 (StataCorp LLC).

We examined yearly RTI episodes and patient and consultation characteristics distribution. We visualised monthly RTI episodes and the distribution of antibiotic treatment types for RTIs using population weighted rates. We chose to report RTI episode rates per 1000 inhabitants to facilitate comparison between different settings.

We estimated the association between pandemic period and antibiotic treatment for RTI episodes using log-binomial regression and reported relative risk (RR) and 95% confidence interval (CI). The analysis was stratified by age group, service type and consultation mode separately. In addition, we analysed the data in the same way, while excluding COVID-19-specific diagnoses, to estimate the effect of the pandemic on non-COVID-19 related RTIs. Service type and consultation mode were stratified by number of consultations per episode, to account for ‘mixed’ episodes necessarily having two or more consultations.

The distribution of antibiotic treatment types during the first month of lockdown (12 March to 12 April 2020) was compared with the same period in the other years by a Chi-squared test. We used the previously defined antibiotic types and subdivided macrolides into azithromycin (J01FA10) and other macrolides.

For consultations initially in the pandemic, we provided charts for daily rates of RTI consultations and antibiotic treatment, service type and consultation mode, from 1 February2020 to 30 April 2020.

### Ethics

Data was acquired in pseudonymised form, stored and analysed on restricted access research servers. The study was approved by the regional ethics committee (REC West, reference number: 135576) and was exempt from requiring informed consent from participants.

## Results

### Study sample

From 2018–2021 there were 4 904 376 RTI episodes, containing 6 707 781 RTI consultations and 1 139 217 antibiotic treatments, spanning 2 511 178 individual patients.

### RTI episode characteristics

Despite a slightly higher proportion of RTI episodes with OOH or mixed service types in 2020 and 2021 compared to 2018 and 2019, the proportion of RTI episodes handled exclusively in daytime general practice was still approximately 80% (Table 1). There was an increase in the proportion of RTI episodes with electronic consultations over the study period from less than 1% in 2018 to more than 50% in 2021.

**Table 1.** Respiratory tract infection episodes per 1000 inhabitants and patient characteristics.

	Year (index consultation)			
	2018	2019	2020	2021
*N*	245 (100%)	235 (100%)	214 (100%)	222 (100%)
Antibiotic treatment				
No	183 (74.7%)	174 (73.9%)	182 (84.7%)	190 (85.4%)
Yes	62 (25.3%)	62 (26.1%)	33 (15.3%)	32 (14.6%)
Patient age				
0–4	681 (26.1%)	663 (26.4%)	425 (20.0%)	592 (25.9%)
5–14	208 (8.0%)	190 (7.5%)	179 (8.4%)	199 (8.7%)
15–24	330 (12.7%)	330 (13.1%)	262 (12.3%)	300 (13.1%)
25–34	223 (8.5%)	223 (8.9%)	262 (12.3%)	260 (11.4%)
35–44	213 (8.2%)	200 (8.0%)	235 (11.1%)	243 (10.6%)
45–54	188 (7.2%)	178 (7.1%)	189 (8.9%)	191 (8.3%)
55–64	200 (7.7%)	187 (7.4%)	174 (8.2%)	162 (7.1%)
65–74	185 (7.1%)	177 (7.0%)	137 (6.4%)	120 (5.2%)
75–84	196 (7.5%)	187 (7.4%)	135 (6.4%)	112 (4.9%)
85+	183 (7.0%)	180 (7.1%)	130 (6.1%)	109 (4.8%)
Patient sex				
Male	215 (43.7%)	205 (43.6%)	185 (43.2%)	196 (44.2%)
Female	276 (56.3%)	266 (56.4%)	244 (56.8%)	248 (55.8%)
Service type				
DGP	206 (84.1%)	198 (84.2%)	170 (79.3%)	182 (82.0%)
OOH	27 (11.1%)	26 (11.1%)	30 (14.0%)	26 (11.6%)
Mixed	12 (4.8%)	11 (4.7%)	14 (6.7%)	14 (6.4%)
Consultation mode				
In-person	244 (99.3%)	232 (98.7%)	127 (59.1%)	110 (49.4%)
Electronic	1 (0.3%)	1 (0.6%)	67 (31.2%)	87 (39.3%)
Mixed	1 (0.4%)	2 (0.7%)	21 (9.7%)	25 (11.3%)

DGP: Daytime general practice. OOH: Out-of-hours.

### Change in antibiotic treatment rate for RTIs

The antibiotic treatment rate for RTIs during the COVID-19-pandemic halved compared to the pre-pandemic period (RR 0.52, 95% CI 0.52–0.52) (Table 2). There was a reduction across all age groups and all service types, except in RTI episodes with a single electronic consultation.

When analysing only non-COVID-19 RTI diagnoses from the analysis, there was still a reduced antibiotic treatment rate for RTIs (RR 0.74, 95% CI 0.74–0.74) (Supplementary Table 1).

**Figure 1.**
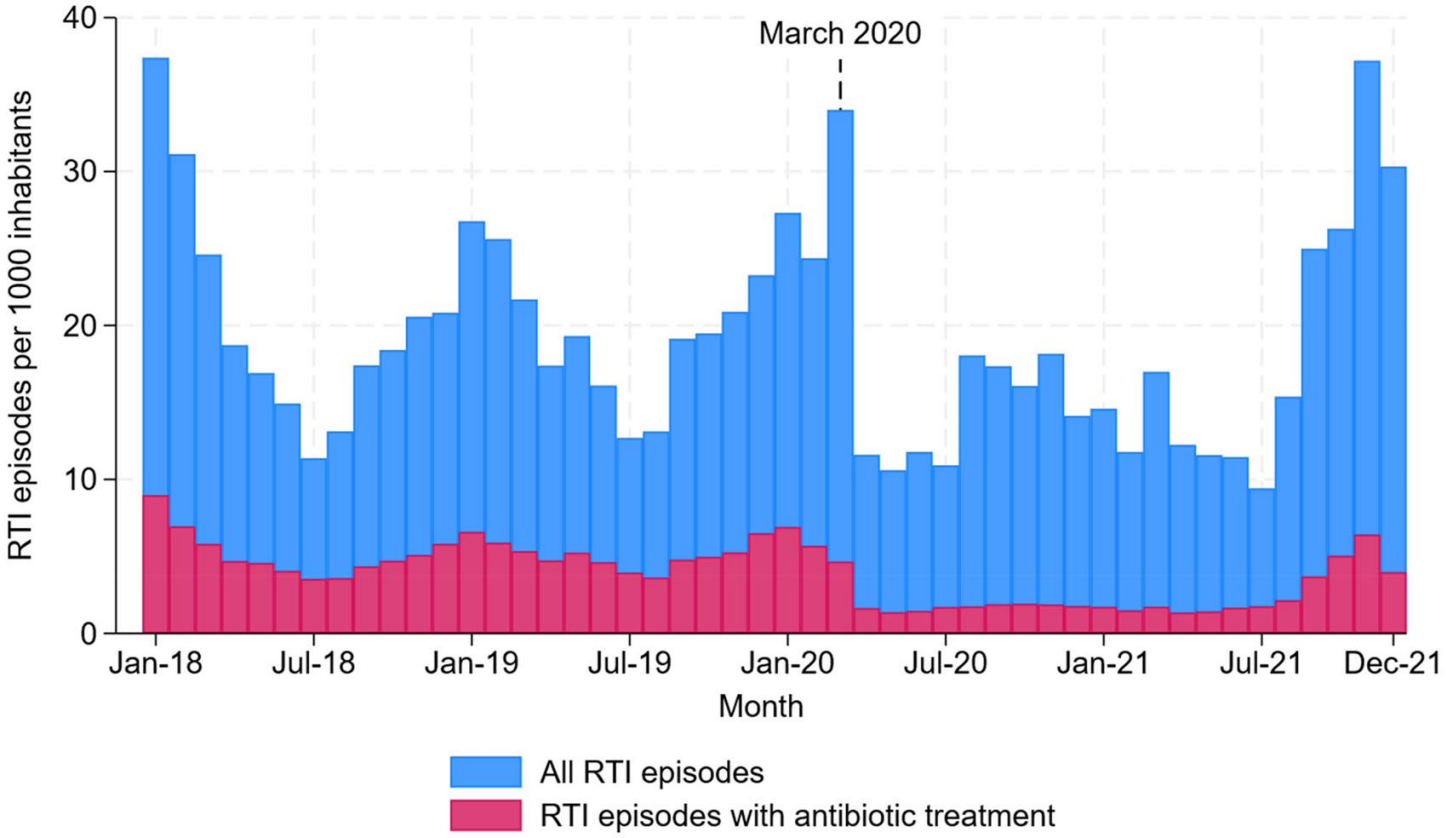
Monthly respiratory tract infections in Norwegian primary care (2018–2021).

Most RTI episodes were without antibiotic treatment. Seasonal variations were present in both total RTI episodes, and RTI episodes with antibiotic treatment pre-pandemic, but such seasonal variations were less prominent during the pandemic period. After March 2020, there was a clear decrease in the rate of RTI episodes (Figure 1). The low rates of RTI episodes in total, and of RTI episodes with antibiotic treatment, persisted until the latter part of 2021, when they both increased.

There was a clear spike in the number of RTI consultations during the initial phase of the pandemic on and around 12 March 2020, which was almost exclusively made up of DGP consultations without antibiotic use. Additionally, the proportion of electronic RTI consultations increased massively (Supplementary Figure 1).

### Antibiotic type distribution

**Figure 2.**
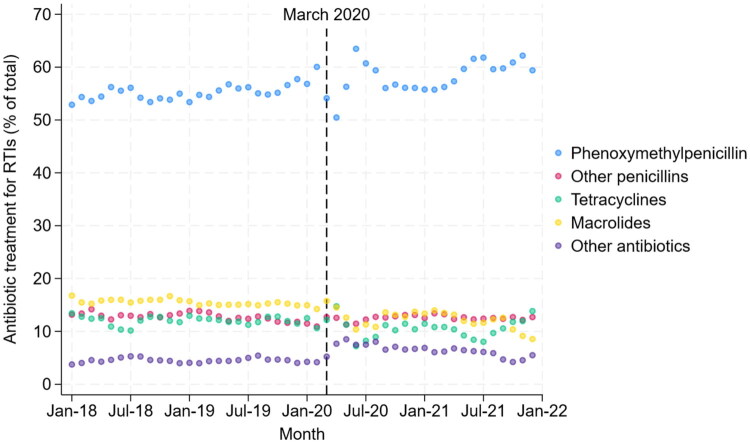
Antibiotic treatment types for respiratory tract infections in Norway (2018–2021).

There was a clear drop in antibiotic treatment for RTIs in March 2020 (Figure 1). Towards the end of 2021, antibiotic treatments for RTIs increased again, more resembling pre-pandemic levels. Over the whole study period, phenoxymethylpenicillin accounted for over half of all antibiotic treatments for RTIs, and the distribution of antibiotic types was relatively stable with some changes during the initial months of the pandemic (Figure 2).

There was a slight decrease in the proportion of azithromycin use for RTIs during the first month after the initial lockdown in 2020 (3.2%) as compared to the corresponding month in 2018 (3.7%) and 2019 (3.7%) (Table 3).

**Table 3.** Antibiotic treatments for respiratory tract infections 12 March to 12 April (2018–2021).

	Year									
	2018		2019		2020		2021		Total	
	*n*	%	*n*	%	*n*	%	*n*	%	*n*	%
Antibiotic type										
Azithromycin	1327	3.7	1276	3.7	663	3.2	318	3.3	3584	3.5
Other macrolides	4250	11.8	4091	11.8	2617	12.7	979	10.0	11937	11.8
Phenoxymethylpenicillin	19430	53.8	18892	54.4	10569	51.2	5551	56.9	54442	53.8
Other penicillins	4921	13.6	4600	13.3	2778	13.5	1280	13.1	13579	13.4
Tetracyclines	4583	12.7	4278	12.3	2719	13.2	1023	10.5	12603	12.5
Other antibiotics	1595	4.4	1573	4.5	1299	6.3	612	6.3	5079	5.0
Total	36106	100	34710	100	20645	100	9763	100	101224	100

*n*: Number of treatments. %: Percentage of total antibiotic treatments.

Percentages calculated by column.

Pearson Chi-squared test for association between year and antibiotic type: *p* < 0.0001.

The proportion of macrolides, other than azithromycin, used for RTIs increased in the first month of lockdown in 2020 (12.7%) compared to in the corresponding month in 2018 (11.8%) and 2019 (11.8%) but decreased in 2021 to lower than pre-pandemic years (10.0%).

## Discussion

### Summary

We observed a large reduction in both the number of RTI episodes and the proportion of RTI episodes with antibiotic treatment, in primary care during the COVID-19 pandemic. Most RTI episodes were handled in DGP, and the sudden increase in RTI consultations in the first month of the pandemic was almost exclusively handled in DGP. The distribution of antibiotic types was relatively stable throughout the study period, even for azithromycin, with over half of antibiotic treatments for RTIs being phenoxymethylpenicillin.

### Strengths and limitations

A major strength of this study is the scope and completeness of the data, comprised of nearly all consultations in Norwegian primary care and all dispensing of oral systemic antibiotics from pharmacies in the 4-year study period. As an observational registry study, this study is well suited to describe tendencies and trends in the data material but does not provide causal associations.

A limitation is the lack of data from a longer pre- and post-pandemic period. A longer observation period would have strengthened the analyses but was unfortunately not made available for the project.

We chose our definition of RTIs to achieve high sensitivity. As COVID-19 is a viral infection for which the guidelines do not recommend antibiotic treatment, the inclusion of COVID-19-specific diagnoses in the RTI episode definition could have led to lower rates of antibiotic treatment. When excluding COVID-19-specific diagnoses, we found that the decrease in antibiotic treatment rate for RTIs was smaller, but still substantial.

This could indicate a reduction in inappropriate antibiotic treatment for RTIs, even non-COVID-19 RTIs. Other explanations for the reduced antibiotic treatment rate could be changes in coding behaviour among physicians when prescribing antibiotics, or an increase in consultations where antibiotics were not required or requested.

Our definition of antibiotic treatment was based on the temporal link between an RTI consultation and antibiotic dispensing. This may have caused an overestimation of antibiotic treatment rates. However, this will not have impacted the observed change in antibiotic treatment rate over time. Furthermore, as RTIs are the most common reason for antibiotic treatment in primary care and we excluded antibiotics used solely for urinary tract infections, the overestimation would likely be negligible.

We did not include telephone contacts as they are usually of an administrative nature. Furthermore, telephone contact diagnosis codes are increasingly non-specific, making them less useful for this kind of research [20]. On the other hand, if telephone contacts were for follow-ups, and led to antibiotic treatment more than 7 days after the face-to-face RTI consultation, this would have been missed in the study, underestimating the antibiotic treatment rates for RTIs.

We observed a massive increase in the use of electronic consultations in RTI episodes during the pandemic. Investigating changes in antibiotic treatment rate for electronic RTI episodes is limited by the small number of pre-pandemic electronic RTI episodes, as these likely represent very early adopters.

It remains uncertain whether the surge in consultations during the first month following lockdown reflects a true increase in treatment-requiring RTIs. This rise may partly be a result of a sudden shift in health care seeking behaviour, driven by heightened uncertainty in the general population and a lowered threshold for contacting DGP and OOH services for symptoms that would not typically have led to consultations. Additionally, physicians’ coding practices may have changed due to increased pressure on the health care system, with RTI diagnosis codes used more frequently, even for consultations primarily involving counselling of patients concerned about the emerging pandemic. This may have contributed to an overestimation of RTI episodes without antibiotic treatment during this first month post-lockdown.

### Comparison with previous literature

Our finding of a decrease in antibiotic treatment for RTIs during the pandemic, and a clear increase in the use of electronic consultations for RTIs in 2020 and 2021, is in line with findings from other European countries [10].

The ability of DGP to adapt to the increased number of consultations during the COVID-19 pandemic was also seen during the 2009 influenza pandemic, showing that GPs are still flexible and able to provide substantial national pandemic preparedness [21]. A study from Dutch primary care found a reduction in RTI episodes treated with antibiotics from before the COVID-19 pandemic (21%) to the pandemic period (13%) [18]. Although the Dutch study had a lower pre-pandemic antibiotic treatment rate than our study, the pandemic antibiotic treatment rate in the studies were similar.

In a study from Danish primary care, a reduction in antibiotic use during the pandemic was found, with the distribution of antibiotic types being similar pre-pandemic and pandemic [22]. This is consistent with our findings, but it is important to note that the Danish study did not investigate RTIs specifically and found a somewhat different distribution of antibiotic types.

The apparent disruption of seasonality in antibiotic treatment of RTIs is in line with a study in Spanish primary care [23]. While this was not a major result in our study, it can strengthen the generalisability of our study.

### Interpretation

The reduction in RTI episode incidence during the pandemic period is likely explained by the shift in societal adaptation to the pandemic, such as infection prevention measures and changes in health care organisation. This would explain both the reduction in primary care RTI episodes, and the overall reduction in the number of antibiotics used.

Furthermore, one may assume that the decrease in RTI episodes meant that only patients with more severe RTIs sought care during the pandemic, which could have increased the antibiotic treatment rate. On the contrary, we found a reduction in antibiotic treatment for RTI episodes, including non-COVID-19-RTIs. We therefore believe that the findings also support an interpretation that GPs uphold their duty of antimicrobial stewardship during crises, while adapting to surges in health care seeking behaviour such as around the initial lockdown. This highlights the resilience of primary care, its important role as the first point of contact with the health care system, and its value for future emergency preparedness efforts.

## Conclusion

There was a sharp reduction in the antibiotic treatment for RTIs in Norwegian primary care during the COVID-19 pandemic, due to both fewer RTI episodes and lower antibiotic treatment rates for RTIs. Types of antibiotic treatment for RTIs barely changed, and we found no evidence of azithromycin being widely used for RTIs immediately following lockdown in March 2020.

## Supplementary Material

Supplemental Material
