# Supplementary material for: Impact of the COVID-19 pandemic on antibiotic treatment for respiratory tract infections in Norwegian primary care
Source: Scand J Prim Health Care. 2026 Jan 19;44(1):2617522. doi: 10.1080/02813432.2026.2617522 (PMC12818330; doi:10.1080/02813432.2026.2617522)

# Supplementary table 1:

| Supplementary table 1: Non-COVID-19 respiratory tract infection episodes and antibiotic treatment before and during the COVID-19 pandemic (N=3253157). | | | | | | |
| --- | --- | --- | --- | --- | --- | --- |
|  | Pre-pandemic | | Pandemic | | Risk ratio | 95 % CI |
|  | No. of episodes | % with antibiotic treatment | No. of episodes | % with antibiotic treatment |  |  |
| Patient age |  |  |  |  |  |  |
| 0–4 | 310384 | 21.1 | 179400 | 16.4 | 0.78 | 0.77–0.79 |
| 5–14 | 192965 | 24.0 | 111698 | 15.1 | 0.63 | 0.62–0.64 |
| 15–24 | 357801 | 24.8 | 180686 | 24.7 | 1.00 | 0.99–1.01 |
| 25–34 | 263420 | 26.1 | 192047 | 17.4 | 0.67 | 0.66–0.67 |
| 35–44 | 226333 | 27.3 | 165033 | 17.6 | 0.65 | 0.64–0.65 |
| 45–54 | 213818 | 26.5 | 140048 | 18.0 | 0.68 | 0.67–0.69 |
| 55–64 | 189547 | 29.0 | 115487 | 20.7 | 0.71 | 0.70–0.72 |
| 65–74 | 151990 | 32.0 | 80049 | 23.8 | 0.74 | 0.73–0.75 |
| 75–84 | 84289 | 31.7 | 47132 | 23.7 | 0.75 | 0.73–0.76 |
| 85+ | 33520 | 32.6 | 17510 | 24.4 | 0.75 | 0.73–0.77 |
| Total | 2024067 | 26.1 | 1229090 | 19.3 | 0.74 | 0.74–0.74 |
| Service type* |  |  |  |  |  |  |
| DGP (1) | 1418207 | 19.4 | 759938 | 12.8 | 0.66 | 0.66–0.67 |
| OOH (1) | 210610 | 29.3 | 120849 | 26.0 | 0.89 | 0.88–0.90 |
| DGP (2+) | 287219 | 46.1 | 231018 | 27.3 | 0.59 | 0.59–0.60 |
| OOH (2+) | 14016 | 47.0 | 11558 | 44.4 | 0.94 | 0.92–0.97 |
| Mixed (2+) | 94015 | 56.5 | 105727 | 37.8 | 0.67 | 0.66–0.67 |
| Consultation mode* |  |  |  |  |  |  |
| In-person (1) | 1619923 | 20.7 | 558652 | 17.3 | 0.83 | 0.83–0.84 |
| Electronic (1) | 8894 | 5.9 | 322135 | 10.1 | 1.71 | 1.57–1.86 |
| In-person (2+) | 382737 | 48.9 | 103295 | 39.9 | 0.82 | 0.81–0.82 |
| Electronic (2+) | 663 | 10.9 | 72671 | 16.8 | 1.55 | 1.24–1.93 |
| Mixed (2+) | 11850 | 39.6 | 172337 | 31.8 | 0.80 | 0.78–0.82 |
| *Parentheses indicate number of consultations in episode. CI: Confidence interval. DGP: Daytime general practice. OOH: Out-of-hours. Mantel-Haenszel test for heterogeneity, p-values for differences in patient age: < 0.0001; service type: < 0.0001; consultation mode: < 0.0001. | | | | | | |

# Supplementary table 2

| Supplementary table 1: Selection of respiratory tract infection diagnosis codes from the International Classification of Primary Care 2 (ICPC-2) | |
| --- | --- |
| ICPC-2 code | Description |
| H01 | Ear pain/earache |
| H70 | Otitis externa |
| H71 | Acute otitis media/myringitis |
| H72 | Serous otitis media |
| H74 | Chronic otitis media |
| R05 | Cough |
| R09 | Sinus symptom/complaint |
| R21 | Throat symptom/complaint |
| R25 | Sputum/phlegm abnormal |
| R71 | Whooping cough |
| R72 | Strep throat |
| R74 | Upper respiratory infection acute |
| R75 | Sinusitis acute/chronic |
| R76 | Tonsillitis acute |
| R77 | Laryngitis/tracheitis acute |
| R78 | Acute bronchitis/bronchiolitis |
| R80 | Influenza |
| R81 | Pneumonia |
| R83 | Respiratory infection other |
| R991* | COVID-19 (suspected/probable) |
| R992* | COVID-19 (confirmed) |
| *COVID-19-specific diagnoses | |

**Supplementary figure 1:** Daily respiratory tract infection (RTI) consultations by antibiotic treatment type, service type, and consultation mode (March–April 2020)

**Alt text:** Three bar charts labelled A to C showing a temporary increase in consultations after March 12. Part A shows an increase in consultations, but not antibiotic treatment. B shows the increase was in daytime general practice, not out-of-hours services. C shows the increase was mostly electronic consultations, not in-person.


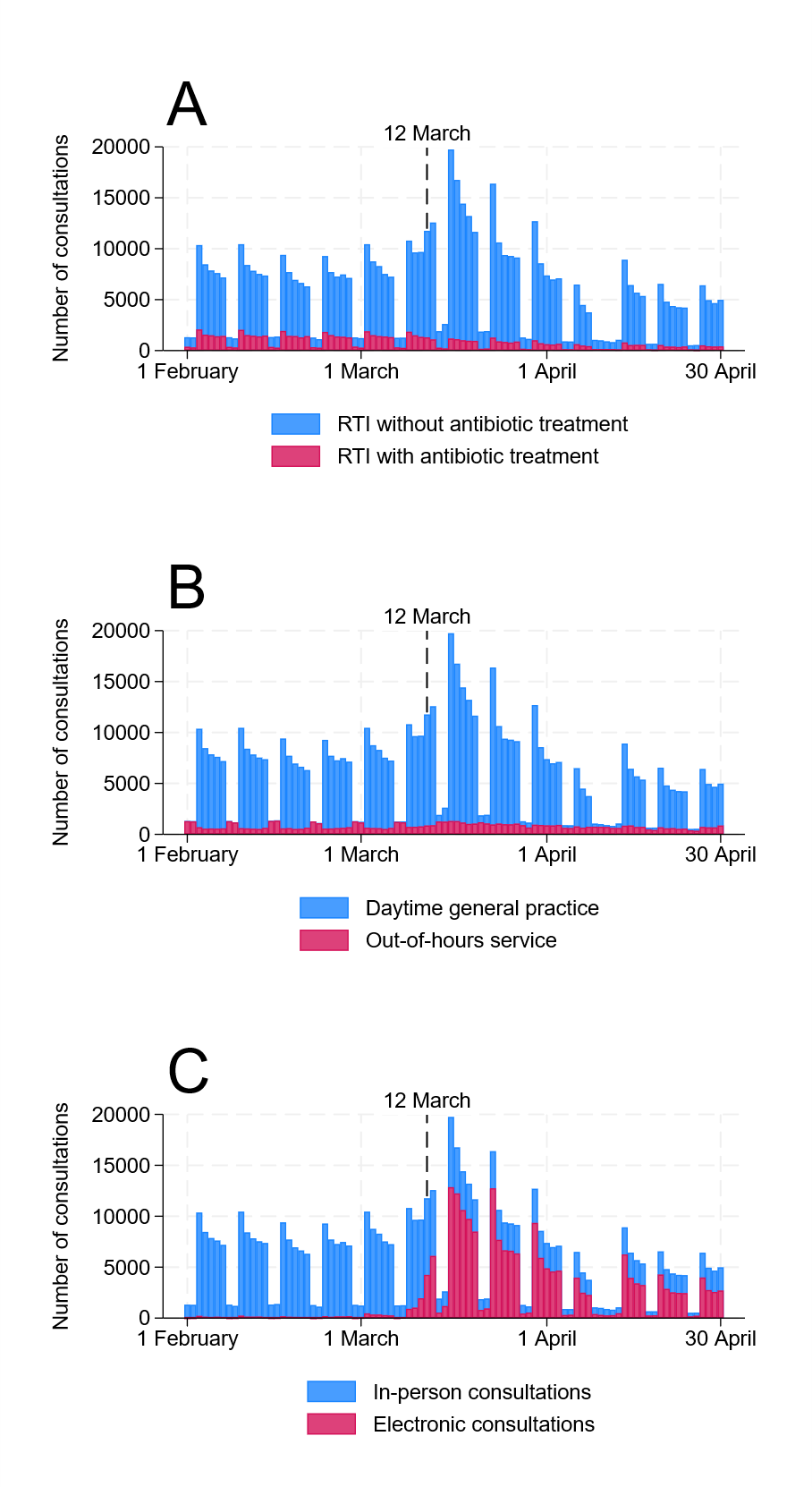

Supplement: Supplemental Material [file IPRI_A_2617522_SM5287.docx]
